# Development and Evaluation of the Delivery-Based HEALED Produce Rx Program for Uninsured Patients With Diabetes in Rural Eastern North Carolina

**DOI:** 10.5888/pcd20.220384

**Published:** 2023-06-22

**Authors:** Lauren R. Sastre, Brandon Stroud, Elisa Smith, Khadijah Hendrix, Olivia McBride

**Affiliations:** 1 Department of Nutrition Science, College of Allied Health Sciences, East Carolina University, Greenville, North Carolina

## Abstract

Produce prescription (PRx) programs have emerged as a preventive treatment to subsidize the cost of fruits and vegetables for people with lower income and have shown promise in improving diet quality and diabetes-related health outcomes (eg, glycated hemoglobin A_1c_). Researchers from the Department of Nutrition Science at East Carolina University worked with the Wayne Action Teams for Community Health (WATCH) Clinic, a safety-net clinic in rural Eastern North Carolina, and a local research farm to develop a PRx program for rural patients with type 2 diabetes and no health insurance. Preliminary patient surveys identified high levels of interest in a PRx program and a desire for recipes to accompany the produce. Formative evaluation results via telephone interviews with eligible patients identified transportation barriers to participation and the desire for complementary nutrition education and culinary resources. These results led to a delivery-based PRx program implemented from June through November 2021. Patients received weekly home delivery of an average of 4.7 pounds of fruits and vegetables and complementary nutrition and health education materials and culinary resources (cookbook, recipes). The level of patient satisfaction with the program was high; the reported level of consumption of produce, including unfamiliar produce, was high; educational resources were associated with increased knowledge and motivation to make healthful lifestyle changes, and glycemic control significantly improved. Ensuring that patients have a voice in the design and implementation of PRx programs is crucial to success. Ongoing use of rigorous formative and process evaluations can ensure appropriateness, use, and a positive effect of PRx programs, and they are needed to establish best practices for implementation.

**Summary**

**What is already known on this topic?**

Produce prescription (PRx) programs have potential to improve glycemic control for people with diabetes.

**What is added by this report?**

Delivery-based PRx programs can address transportation barriers for underresourced participants in large rural counties by alleviating the need for participants to find transportation to a farmers market or local retailer. Nutrition, health, and culinary educational resources are desirable and effective in PRx programs.

**What are the implications for public health practice?**

Ongoing use of rigorous formative and process evaluations can ensure appropriateness, use, and effect of PRx programs, and are needed to establish best practices for PRx implementation.

## Introduction

One in 10 people in the US has been diagnosed with diabetes; most (90%–95%) are diagnosed with type 2 diabetes (hereinafter, diabetes) (1). Maintaining glycemic control is essential to limit diabetes-related complications and comorbidities and can be optimized through adherence to medications and improved diet quality (2,3). Although dietary changes can have a positive effect on glycemic control, a lack of access to healthy food can impede control (4).

One emerging approach to improve healthy food access and diet quality is the provision of a produce prescription (PRx), which is defined as “a medical treatment or preventative service for patients . . . due to a diet-related health risk or condition, food insecurity . . . and are referred by a healthcare provider or health insurance plan” (5). PRx programs have become more prevalent since the expansion of the Gus Schumacher Nutrition Incentive Program and the allocation of $25 million in funding for PRx programs from 2019 through 2023 (5). PRx programs have demonstrated effectiveness in supporting improved glycemic control (6,7).

Although PRx programs are promising, they can be improved. First, PRx programs commonly use tokens or vouchers, which can be associated with social welfare stigma, and participants typically must drive to an additional and potentially less familiar location (eg, farmers market) to redeem them (5–9). Second, additional shortcomings are a lack of program implementation in southern and rural areas, short program duration, and a limited monetary value for vouchers (5,9). In addition, participation rates have been low in rural areas (as low as 13.5%) (9). PRx programs that address participation barriers are needed in rural, southern populations. To address these gaps, an academic–agricultural–clinical partnership was formed among the Department of Nutrition Science at East Carolina University, an agricultural research farm, and the Wayne Action Teams for Community Health (WATCH) Clinic to develop, implement, and evaluate a PRx program tailored for people with lower income, diabetes, and no health insurance in rural eastern North Carolina.

## Purpose and Objectives

The research team worked with clinic staff and health care providers, including a physician, a nurse, and a case worker, who had knowledge and experience in working with this unique patient population to develop and validate the content of a patient survey. Trained student research assistants collected survey data on iPads from patients via before or after clinic visits in the waiting room of the clinic from February through May 2019. The study team used SPSS version 28.0 (IBM Corp) and descriptive statistics for analysis. Results from this patient survey (N = 185) indicated strong interest in receiving fresh produce (89.0%) and recipes (85.0%), attending taste testing and cooking workshops (73.6%), and obtaining home gardening support (56.3%). Few survey participants indicated receiving food assistance (Supplemental Nutrition Assistance Program [SNAP], 39.1%; food pantry 11.7%). On the basis of this information, the study team implemented an 8-week food recovery–based PRx pilot program in summer 2019; 30 patients with diabetes or hypertension were enrolled and given weekly produce and recipes (10). This early PRx initiative confirmed transportation as a consistent barrier (10,11). To guide development of a more robust PRx program, the study team conducted a formative evaluation, which led to a 24-week delivery-based PRx program, HEALED: Healthy Eating and Active Lifestyle to Enhance Diabetes management. The objective of this study was to evaluate 1) the use, barriers, and impact of the produce and health education resources provided by the program and 2) the experience, perceptions, and glycemic control of HEALED participants.

## Intervention Approach

The HEALED PRx program was designed as a pre–post study. It gave participation priority to patients with diabetes and no health insurance because of the high prevalence of diabetes in the clinic population and the region, regional disparities in diabetes, and the lack of access to diabetes resources and programming for uninsured patients in the region. Patients were recruited by telephone and through referrals from on-site clinical administrative staff and health care providers, including a nurse practitioner and a physician’s assistant in May and June 2021. The sample size was determined by a G*Power analysis (12,13), which indicated that a sample size of 34 was necessary to achieve adequate power (0.80) with an effect size of 0.25 and a type I error of 0.05. We oversampled (n = 40 participants) to account for a maximum possible attrition rate of 20% (34 × 1.20 = 40.8); 20% is an established threshold to reduce bias in this type of analysis (14). At an enrollment appointment (May–June 2021), participants completed a preprogram survey, had glycated hemoglobin A_1c_ (HbA_1c_) measurements taken, and received a notebook to store weekly educational handouts and recipes and a $20 reloadable Greenphire ClinCard. At the end of the program, participants received $35. Written informed consent was obtained at enrollment. The study was approved by the University and Medical Center Institutional Review Board at East Carolina University.

The PRx included a mix of familiar and specialty vegetables (exotic varieties, similar size and shape to nonexotic varieties but often different colors) from a local agricultural research farm. Produce varied by season and was selected by the farm’s director for research purposes rather than profit. Produce was harvested, packaged, and delivered to patients’ homes every Friday for 24 weeks from June through November 2021 by student volunteers. Student volunteers completed 3 delivery routes, which took on average 3 to 4 hours in this large rural county and were created by using Routific (https://routific.com). Because the program was implemented during the height of the COVID-19 pandemic, deliveries were contactless. Health education handouts and recipes were provided each week (Table 1). The principal investigator (L.R.S.), a registered dietitian nutritionist, identified topics, and undergraduate research assistants further developed them. Resources were tailored for people with lower income and literacy levels and were based on recommendations by the American Diabetes Association and regional food preferences.

**Table 1:** Health and Nutrition Education Handouts and Produce Delivered During the 24-Week HEALED Produce Rx Program for Low-Income, Rural Patients With Diabetes, Eastern North Carolina, June–November 2021

Program week	Health and nutrition education handout topics	Produce type delivered	Total no. of pounds per participant
1	Goal setting, finding your motivation, social support	Asian sukoy, black radish, radish greens, Brentwood lettuce	5.1
2	5%–10% Weight loss can improve your health	Asian sukoy, black radish, radish greens, Brentwood lettuce, kale	6.8
3	Physical activity — benefits for type 2 diabetes and overall health	Black radish, radish greens, Brentwood lettuce, kale, purple beans	6.0
4	Portion sizes	Black radish, Brentwood lettuce, blueberry, purple beans, kale	5.7
5	Balance/how to build a healthy plate with diabetes	Bush beans, black radish, blueberry, cucumbers, kale, Brentwood lettuce	7.5
6	Understanding carbohydrates/sources of carbohydrates	Cucumbers, black radish, kale	5.5
7	Incorporating more fruits/vegetables	Kale, black radish, peppers, cucumbers	5.3
8	Nonstarchy vegetables: recipes, health benefits	Kale, beans, tomatoes, peppers, cucumbers	3.9
9	Healthy snacks	Okra, sweet peppers, bush beans, tomatoes, cucumbers, kale	3.6
10	Macronutrients	Peppers, okra, tomatoes, melon	3.6
11	Reading food labels	Tomatoes, cucumbers, peppers, okra	5.0
12	Fiber and nutrient dense foods	Okra, peppers	2.2
13	Budget meals and shopping	Okra, peppers	1.9
14	Cooking with frozen and canned vegetables	Okra, peppers	1.3
15	How to maintain lifestyle changes	Melon, peppers, okra, bok choy	3.2
16	Healthy eating on the go	Peppers, okra, bok choy	2.0
17	Slow/batch cooking	Peppers, okra, bok choy, bush beans	1.9
18	2020 Dietary Guidelines overview	Peppers, okra, bok choy	1.0
19	DASH (Dietary Approaches to Stop Hypertension) diet	Peppers, okra	0.8
20	Sodium and potassium electrolyte balance	Peppers, okra, eggplant, daikon radishes, sweet potatoes	6.8
21	Hydration and reducing sugar in drinks	Green beans	2.0
22	Cardiovascular/physical activity guidelines and suggestions	Green peppers, sweet potatoes	12.7
23	Sleep and stress	Sweet potatoes	10.0
24	Carbohydrate counting	Sweet potatoes	10.0

## Evaluation Methods

### Formative evaluation: eligible patient’s interests, barriers, and preferences to optimize the impact of the HEALED PRx program

The research team conducted a formative evaluation via semistructured telephone interviews in December 2020 and January 2021. The interview guide was developed by the principal investigator, and the content was reviewed and validated by 7 nutrition researchers with experience in community-based nutrition programming or working with patients who are medically underserved. Patients with diabetes who had attended a retinopathy clinic in the previous year were contacted by telephone and invited to participate. Interviews were conducted until theme saturation was reached (n = 26 participants). All interviews were recorded and transcribed verbatim. We used deductive content analysis as outlined by Elo and Kyngäs and grouped themes into 3 categories: preferences and promoters, resources, barriers and needs to participate in a PRx program (15). Four research team members (L.R.S., E.S., K.H., B.S.) analyzed transcripts independently, and the team reached consensus on all themes.

### Process evaluation: use of food and nutrition education and culinary resources and program satisfaction

The research team collected weekly surveys via a QR (quick response) code on a reminder sheet or follow-up text messages via Twilio with a survey link. Weekly surveys examined use of the delivered produce, health education, and culinary resources. The team also conducted a final survey on program experience and satisfaction in November 2021. All survey tools used HIPAA-(Health Insurance Portability and Accountability Act) secure Research Electronic Data Capture (REDCap) (16).

### Impact evaluation: dietary and clinical impact

A preprogram survey and postprogram survey assessed participants’ fruit and vegetable consumption via an all-day intake screening tool from the National Cancer Institute’s Eating at America’s Table Study (17). HbA_1c_ was measured by clinical staff at enrollment (May–June 2021) and at the end of the program (November 2021); these data were then collected by the study team via retrospective medical record review and stored in REDCap (16). The research team used SPSS version 28.0 (IBM Corp) to analyze data and descriptive analysis and paired-sample *t* tests to identify significant changes (*P* < .05) in HbA_1c_.

## Results

### Formative evaluation

The formative evaluation revealed that all 26 participants were interested in participating in a PRx program. Most patients acknowledged that vegetables were important, yet they were uncertain about their specific nutritional benefits and foods to best manage their diabetes. Nutrition education on specific vegetables or recipes and cooking or taste testing were desired. Reported barriers included limited transportation and complicated schedules. Barriers to consuming produce included family food preferences (dislike of vegetables), a lack of time (busy or constrained schedules), and a lack of motivation to cook. These results were used to prioritize a delivery-based approach, the development of simple, quick recipes, and health education handouts with a focus on nutrition and other healthy lifestyle habits.

### Process evaluation

Forty participants enrolled in the intervention; most (n = 27) were women, 22 were non-Hispanic White, 18 were non-Hispanic African American, and the mean (SD) age was 54.8 (6.6) years. Half (n = 20) were employed and most (n = 25) had an annual household income at or below $29,999. Twenty-one participants completed the weekly process evaluation surveys, and 8 participants completed 5 or more of these surveys. Weekly survey responses showed that respondents used three-quarters or all of the produce each week and the primary barriers to use were unfamiliarity, dislike, or uncertainty about how to cook. Participants reported most produce items as familiar. Feedback on the educational handouts indicated half of respondents reviewed the materials, and of those, most agreed that the information was new and motivated them to make changes in nutrition and physical activity. The overall program experience and satisfaction survey revealed high levels of satisfaction; 29 of 32 participants indicated that that they were very satisfied or satisfied (Table 2). The surveys also indicated increased access to produce, improved self-reported diet quality, and willingness to try unfamiliar produce.

**Table 2:** Program Perceptions, Experience, Satisfaction, and Impact Reported by Rural, Uninsured Patients (n = 32) Who Participated in the 24-Week Delivery-Based HEALED Produce Rx Program, Eastern North Carolina, June–November 2021

Survey question	Possible responses				
**How likely would you be to recommend this program to your friends or family?**	**Very likely**	**Likely**	**Neither**	**Not likely**	**Not likely at all**
	23	5	2	1	1
**What was your overall satisfaction with the program?**	**Very high**	**High**	**Neutral**	**Low**	**Very low**
	20	9	3	0	0
**I believe this program . . .**	**Strongly agree**	**Agree**	**Neither**	**Disagree**	**Strongly disagree**
Increased my access to fresh produce.	18	12	0	1	1
Helped me overcome cost barriers to obtaining adequate fresh produce.	16	9	3	4	0
Increased my intake of fruit and vegetables.	16	12	2	2	0
Increased my willingness to try new or unfamiliar produce.	18	10	2	2	0
Helped me improve my overall diet quality.	18	9	1	4	0
Helped me to follow nutrition recommendations given by my medical provider.	13	14	2	3	0
Helped me to control my blood glucose.	13	12	4	2	1
Helped me become more physically active.	8	13	4	4	3
Was informative.	17	12	2	1	0
Was enjoyable.	20	12	0	0	0
**Please rank the following for why you joined the program.**					
Improve my health	21	10	1	0	0
Improve my blood glucose control	21	8	1	2	0
Recommended by my medical provider	20	10	2	0	0
Nutrition information	19	10	2	1	0
Cooking support (knowledge, skills, recipes)	19	8	4	1	0
Improve access to fresh produce	18	10	1	2	1
Lose weight	17	8	4	2	1
**How would you rate the recipes you have used?**	**Very easy to understand**	**Somewhat easy to understand**	**No opinion**	**Somewhat difficult to understand**	**Very difficult to understand**
	22	6	3	0	1
**Did any of the following impact your ability to use/prepare the recipes? (Check all that apply.)**	**Did not have necessary cooking equipment/tools**	**Did not have other ingredients called for in the recipe**	**Unfamiliar with ingredients in the recipe**	**Other**	**—**
	1	4	13	17	

### Impact evaluation

Participants’ median (IQR) preprogram self-reported total fruit and vegetable consumption increased from 1.2 (0.4–2.0) to 1.6 (0.2–3.0) servings per day preprogram to postprogram. Glycemic control significantly improved: we found a mean decrease of 0.47% HbA_1c_ (n = 35) from preprogram (7.63%; SD, 1.63%) to postprogram (7.16%; SD, 1.40%) (*t* = 3.47; *P* = .001).

## Implications for Public Health

The development and implementation of the HEALED PRx program was guided by a needs assessment, pilot programming, and a formative evaluation that identified barriers to participation as well as resource preferences and needs. By addressing barriers, preferences, and needs, the delivery-based program was well received and helped to increase access to healthy food and nutrition education and improve glycemic control in this rural, uninsured, medically underserved population.

The HEALED PRx program had several strengths. HbA_1c_ values among participants declined an average 0.47% from preprogram to postprogram. This clinical improvement was less than the change (−0.8%) reported in a previous systematic review and meta-analysis by Bhat et al; however, this decline is clinically and statistically significant (6). The HEALED PRx program also distributed larger quantities of produce (5–7 lb/wk for 24 wk) than likely was achievable by typical voucher programs and ensured access during the COVID-19 pandemic, when food insecurity was heightened (8,9). The HEALED PRx program also addressed barriers (eg, transportation) faced by lower-income, rural populations by delivering produce directly to participants’ homes. Although some produce was unfamiliar, most participants reported consuming most of the produce and a willingness to try other unfamiliar produce because of the exposure during the program. Increased culinary support and more direct nutrition education may complement and optimize the effect of PRx programs on dietary behaviors.

The HEALED PRx program also had limitations. It used donated produce via a local agricultural research farm, and such farms may not be accessible in other regions or sustainable in the long term. Students contributed to the implementation and evaluation of this program and may not be available as stable, long-term resources; however, connections with academic institutions for internships, field placement, and practicum experiences that provide structured support, experiential learning, and professional development for future public health practitioners could be explored. Additionally, although delivery of produce ensured access, a delivery service may be difficult to replicate. Prioritizing patients with the greatest social (eg, food insecurity, lack of transportation resources), nutrition, and health risk factors (eg, poor glycemic control) for delivery may be warranted. Coordination and distribution of produce while participants are on-site for other health or nutrition services may improve access and is used in other PRx programs (18).

It is important to highlight that our study included a specific, southern, rural, medically underserved patient population and was tailored for their needs, and our findings may not be generalizable to the general US population with diabetes. Future PRx programming should identify and address barriers (eg, transportation) to ensure access. Evaluation of the use of the PRx and resources needs to be strengthened, and although participant fatigue set in during the HEALED PRx program (for example, after the first few weeks <25% of the participants completed weekly surveys), process evaluation efforts are critical to evaluate and better understand implementation barriers. A monthly survey may have been better than a weekly survey. Comprehensive evaluation of PRx programs that use rigorous validated tools is critical to identifying optimal approaches to establish best practices for PRx programming to guide and ensure effectiveness.
